# Implementation of a regional stroke reform in Denmark: a tale of cost reduction and quality improvement

**DOI:** 10.1186/1472-6963-14-S2-P30

**Published:** 2014-07-07

**Authors:** Karla Douw

**Affiliations:** 1 Centre for Public Health & Quality Improvement, Aarhus, Denmark

## Background

In May 2012, the Central Denmark Region implemented a reform in stroke care, which included concentration of acute stroke treatment from 5 to 2 specialized hospitals, short stay, and a shift from inpatient rehabilitation to community-based rehabilitation, and establishment of 5 stroke teams. The plan was announced three months before implementation. Patients were promised a more integrated care pathway with newly established stroke teams supporting the discharge and rehabilitation in the home municipality.

In this reform, only hospital care is within the Region’s area of control, as rehabilitation at home is the responsibility of municipalities in this decentralized health care system. The top-down implementation of this reform poses therefore some challenges.

## Methods

A policy-analysis was carried out based on Buse et al.’s health policy triangle and Winter’s model for public policy implementation. Insight in the policy formulation, design and implementation process of the stroke reform was obtained from semi-structured interviews with representatives of the health authority, hospitals, municipalities and general practitioners (n=8). Additionally, an analysis of relevant policy and practice documents was carried out. Data were analysed using thematic analysis.

## Results

Many of the stakeholders have a big interest, but have had little or no influence on the design of the reform. Hospitals were involved in formulating and designing the reform in contrast to municipalities. Patients were not involved at all. In accordance with a top-down approach, the Region delegated the implementation of the changes to a committee of stakeholders. No sanctions or incentives were included in the plan to enhance implementation at the street level. All stakeholders however agree with the rationale for the reform, that the quality of (acute) care for patients would improve, which gives the reform legitimacy. Municipalities however were critical towards the idea of rolling out one model of early discharge teams over the region. Besides, the financial consequences of this model were not made clear to them.

## Conclusions

The reform has a hospital-dominated perspective, as this is the area of control of the Region. The conditions for successful implementation are however not fulfilled in the primary sector, where rehabilitation care has to be provided. The implementation of reforms of this scale, with complex chains of implementing actions and indirect control, asks for policy implementation models to be combined with those for implementation of change in health care, with more emphasis on tailored plans for different target groups at the practice level in health care.

